# Toward the identification of methanogenic archaeal groups as targets of methane mitigation in livestock animalsr

**DOI:** 10.3389/fmicb.2015.00776

**Published:** 2015-07-30

**Authors:** Benoit St-Pierre, Laura M. Cersosimo, Suzanne L. Ishaq, André-Denis G. Wright

**Affiliations:** ^1^Department of Animal Science, South Dakota State University, BrookingsSD, USA; ^2^Department of Animal and Veterinary Sciences, The University of Vermont, BurlingtonVT, USA; ^3^Department of Animal and Range Sciences, Montana State University, BozemanMT, USA; ^4^School of Animal and Comparative Biomedical Sciences, University of Arizona, TucsonAZ, USA

**Keywords:** methanogens, 16S rRNA analysis, herbivores, rumen microbiology, methane mitigation

## Abstract

In herbivores, enteric methane is a by-product from the digestion of plant biomass by mutualistic gastrointestinal tract (GIT) microbial communities. Methane is a potent greenhouse gas that is not assimilated by the host and is released into the environment where it contributes to climate change. Since enteric methane is exclusively produced by methanogenic archaea, the investigation of mutualistic methanogen communities in the GIT of herbivores has been the subject of ongoing research by a number of research groups. In an effort to uncover trends that would facilitate the development of efficient methane mitigation strategies for livestock species, we have in this review summarized and compared currently available results from published studies on this subject. We also offer our perspectives on the importance of pursuing current research efforts on the sequencing of gut methanogen genomes, as well as investigating their cellular physiology and interactions with other GIT microorganisms.

## Introduction

In herbivores, fermentation of feed by mutualistic gastrointestinal tract (GIT) communities of microorganisms is essential for proper nutrition of their hosts (Hungate, 1966). These microbial communities consist of a great number of species from phylogenetically diverse groups, mainly bacteria, archaea, protozoa, and fungi, that are mutually dependent through complex trophic relationships (Wolin, 1979). As a result of the collective activities of these microorganisms, polysaccharides, proteins, and lipids are metabolized into end products such as volatile fatty acids (VFAs) that are assimilated by their host to fulfill their energy needs.

Certain products of microbial fermentation, such as carbon dioxide and methane, are not absorbed by the host and are released into the environment. There are two main concerns over methane emissions by livestock animals. First, they have a negative impact on animal productivity, as this process results in lost energy from the host, which can range between 2 and 12% of an animal’s energy intake (Johnson and Johnson, 1995). Secondly, methane is a much more potent greenhouse gas than carbon dioxide, thus having a greater effect on climate change (Lashof and Ahuja, 1990). Since the continuous growth of the human population is expected to result in an increase in the number of domesticated ruminants, decreasing methane emissions by livestock has become a priority and an integral part of climate control policies (Thorpe, 2008).

Methane is synthesized by obligate anaerobic archaea that share methanogenesis as part of their energy metabolism (Liu and Whitman, 2008). Many methanogenic archaea, or methanogens, use H_2_ and CO_2_ as substrates to synthesize methane, with certain species also capable of metabolizing small organic compounds such as formate, methanol, methylamines, or acetate (Thauer et al., 2008). Although they do not contribute to fulfilling their host’s energy requirements, methanogens play an important role in the GIT of herbivores by maintaining the fermentative performance of the microbial community. By metabolizing H_2_ generated from fermentation of plant polysaccharides, methanogens function as a sink to maintain a low H_2_ pressure, which promotes plant fiber digestion by protozoa and bacteria (Wolin, 1982).

As the only producers of enteric methane, methanogens are responsible for the contribution of livestock industries to climate change (Thorpe, 2008), and have thus become the focus of research toward developing mitigation strategies. Variations in methane emissions according to host and/or diet present an important challenge toward achieving this goal. For instance, an early study reported that gray kangaroos emitted less methane than sheep fed the same diet (Kempton et al., 1976). Similarly, lower levels of methane were observed for camelids compared to ruminant livestock (Pinares-Patino et al., 2003; Dittmann et al., 2014), and Franz et al. (2010) found that methane emissions were higher in sheep compared to ponies. Methane production has been found to increase on higher forage/cellulose diets, especially when comparing grass forage to legume forage (McAllister et al., 1996). In growing beef cattle, methane emissions were not affected by the type of grain fed during backgrounding, but they were found to be lower for corn compared to barley during the finishing phase (Beauchemin and McGinn, 2005). In contrast, the addition of high quality feeds, oils, plant secondary compounds, or microbial modifiers can reduce methane emissions (Lovett et al., 2003; Woodward et al., 2004; Carulla et al., 2005; Puchala et al., 2005; Beauchemin et al., 2007, 2009; Grainger et al., 2009, 2010). In most cases, this variation does not appear to be due to differences in methanogen cell density, but rather in the composition of the methanogen community. For instance, it was observed during anti-methanogen vaccination trials that, while methane emissions from immunized animals were decreased in early stages, they returned to control levels after prolonged immunization (Wright et al., 2004). The vaccine was expected to target methanogens that were highly represented in the rumen microbial community, which may have allowed other methanogens that would otherwise be at a disadvantage to increase in abundance in the rumen of immunized animals (Williams et al., 2009). Based on these results, it was hypothesized that GIT methanogen communities may consist of different groups that could vary in their potential for growth and methane production.

Since the composition of a methanogen community represented a likely determinant of its capacity to produce methane, the investigation of mutualistic methanogen communities in the GIT of a variety of host herbivores or in response to different diets has been the subject of active and ongoing research. As with other fields in environmental microbiology, research on GIT methanogens has benefited greatly from the rapid technological improvements of culture-independent experimental approaches. In this review, we have summarized and compared data available from published studies on the composition and representation of methanogens in the GIT of herbivores. While they tend to be distinct, according to a variety of factors including host breed, species, diet and geographical location, and by mechanisms that remain poorly characterized (Kim et al., 2011), the data also indicate that GIT methanogens form phylogenetic clusters that exhibit a certain degree of overlap among different communities.

## Prevalent Methanogens in the GIT Communities of Herbivores

Archaea have been identified in a wide range of habitats (Liu and Whitman, 2008), forming a large and diverse prokaryotic domain, not only ecologically but also phylogenetically. The majority of currently known archaeal species have been assigned to the phyla Euryarchaeota or Crenarchaeota, but additional phyla have been proposed to account for the high degree of divergence found in certain archaea, including Thaumarchaeota, Nanoarchaeota, Korarchaeota, Parvarchaeota, and Aigarchaeota (Shin et al., 2004; Allers and Mevarech, 2005; Brochier-Armanet et al., 2008, 2011; Nunoura et al., 2011; Rinke et al., 2013; Petitjean et al., 2014; Raymann et al., 2015). All currently known methanogens belong to the phylum Euryarchaeota, which is divided into seven orders (Methanobacteriales, Methanocellales, Methanococcales, Methanomassiliicoccales, Methanomicrobiales, Methanosarcinales, and Methanopyrales), that include 10 families and 31 genera (Liu and Whitman, 2008; Sakai et al., 2008; Paul et al., 2012; Iino et al., 2013). Methanogens have colonized as a group a wide variety of anaerobic environments, including marine and freshwater sediments, soil, and landfills, and are thus not limited to just the GIT of animals.

The 16S rRNA gene is the most commonly used phylogenetic marker for the characterization of bacterial and methanogen communities (Skillman et al., 2006; Rajendhran and Gunasekaran, 2011). Thus, the data we have selected on methanogen composition from the gut of herbivorous animals was generated using 16S rRNA gene clone libraries or next generation sequencing of amplicons. Typically, a minority of the GIT archaeal 16S rRNA gene sequences identified to date are identical to validly characterized methanogens species, while the remaining majority of sequences exhibit a varying degree of relation to methanogen species. Despite their diversity, GIT methanogens group into very distinct phylogenetic clusters of archaea (Kim et al., 2011). In this section, we aim to present the major groups of methanogens that have been identified in the GIT of herbivores.

### *Methanobrevibacter*-Related Archaea

16S rRNA gene sequences closely related to certain species belonging to the genus *Methanobrevibacter* (order Methanobacteriales) are among the most frequently found in GIT samples from livestock animals. While representation can vary according to host species, diet, and/or geographical location, dominance of *Methanobrevibacter-*related archaea reported by a number of different studies is quite striking. Indeed, *Methanobrevibacter*-related methanogens represented more than ∼80% of 16S rRNA gene sequences from hosts ranging from birds [hoatzin (Wright et al., 2009)] and marsupials [wallaby-May sample (Evans et al., 2009)] to pseudo-ruminants [alpaca (St-Pierre and Wright, 2012), Bactrian camel (Turnbull et al., 2011)] and ruminants [buffalo – Mediterranean breed (Franzolin et al., 2012), cattle-New Zealand (Seedorf et al., 2015), dairy cattle (Hook et al., 2009, 2011; King et al., 2011), goats (Cunha et al., 2011), impala (Cersosimo et al., 2015), reindeer-Norway (Sundset et al., 2009a), sheep-Venezuela (Wright et al., 2008), sheep-Scotland (Snelling et al., 2014), sheep-New Zealand (Seedorf et al., 2015), and yak (An et al., 2005)]. In other studies, they represented a lower, but well represented proportion (27–60%) of identified clones or sequence reads in cattle (Whitford et al., 2001; Skillman et al., 2006), reindeer-Svalbard (Sundset et al., 2009b), white rhinoceroses (Luo et al., 2013), Chinese roe deer (Li et al., 2014), and Mehsani water buffaloes (Singh et al., 2015). Since characterized species of *Methanobrevibacter* mainly use H_2_ and CO_2_ as substrates for methanogenesis, it is hypothesized that uncultured *Methanobrevibacter*-related methanogens identified by their 16S rRNA gene sequence are also hydrogenotrophic.

Currently, 15 cultured *Methanobrevibacter* species have been characterized according to the List of Prokaryotic Names with Standing in Nomenclature (LPSN). However, GIT *Methanobrevibacter*-related methanogens from livestock animals tend to be more closely related to either *Methanobrevibacter ruminantium, Methanobrevibacter millerae, Methanobrevibacter gottschalkii*, or *Methanobrevibacter smithii*. While typically found in lower frequency, 16S rRNA sequences with closest identity to either *Methanobrevibacter olleyae, Methanobrevibacter thaueri*, or *Methanobrevibacter wolinii* have also been reported in GIT samples. *Methanobrevibacter boviskoreani* has been the latest addition to the list of cultured rumen methanogens from this group (Lee et al., 2013), with *Methanobrevibacter wolinii* as its closest relative. To our knowledge, *Methanobrevibacter woesei* related methanogens have only been reported in chickens, and 16S rRNA gene sequences from the GIT of herbivores that are related to either *Methanobrevibacter curvatus, Methanobrevibacter cuticularis, Methanobrevibacter oralis, Methanobrevibacter arboriphilus, Methanobrevibacter filiformis*, or *Methanobrevibacter acididurans* have only rarely if ever been identified in this environment.

### SGMT-RO Population Model for *Methanobrevibacter*-Related Methanogens

While it appears that most *Methanobrevibacter*-related GIT 16S rRNA gene sequences tend to be closely related to a limited number of valid *Methanobrevibacter* species, they exhibit a remarkable level of diversity that has been estimated to be in the 100s of species-level operational taxonomic units (OTUs; Kim et al., 2011). Indeed, the level of sequence identity for *Methanobrevibacter*-related 16S rRNA gene sequences can typically vary between 90 and 100% with their respective closest valid methanogens species. Therefore, although GIT methanogens are from similar phylogenetic groups, they appear to form a continuum of species rather than discrete groups (Janssen and Kirs, 2008). However, only a subset of OTUs are identified in each sample, with typically a few OTUs that tend to be more abundant (Wright et al., 2007, 2009; Sundset et al., 2009a,b; Hook et al., 2011; King et al., 2011; Turnbull et al., 2011; Franzolin et al., 2012; St-Pierre and Wright, 2012; Snelling et al., 2014; Cersosimo et al., 2015; Seedorf et al., 2015). To facilitate the creation of GIT methanogen community structure models from environmental samples, sequence identity cutoffs can be set at specific levels to group 16S rRNA genes from methanogens of the same presumptive species or of the same presumptive genus. The representation of each category in an environmental sample can thus be expressed as a percentage of the total number of clones or sequence reads identified in its corresponding study. Methanogen communities can then be compared between host breeds, species, feed regimens, and/or geographical locations. While there is currently no absolute 16S rRNA gene sequence identity cutoff that has been set to formally distinguish methanogens of the same species or genus from uncultured archaea, it remains a very useful tool to uncover various trends in archaeal community composition.

As a complementary approach, we have also explored the use of phylogenetic analyses of *Methanobrevibacter*-related GIT 16S rRNA gene sequences to create community structure models. While they appear to form a continuum of species, we observed that *Methanobrevibacter*-related GIT 16S rRNA gene sequences are mostly distributed between two large clades. One clade consists of sequences that are closely related to *Methanobrevibacter smithii, Methanobrevibacter gottschalkii, Methanobrevibacter millerae* or *Methanobrevibacter thaueri*, which we have referred to as the smithii – gottschalkii – millerae – thaurei clade, or simply as the SGMT clade. The other major clade groups *Methanobrevibacter ruminantium* and *Methanobrevibacter olleyae* – like sequences, which we have referred to as the ruminantium – olleyae or RO clade.

After re-examining available data by our research team and other research groups to compare the sequence distribution between the SGMT clade and the RO clade, we were able to group samples from a wide variety of sources into more encompassing categories (**Table 1**). For instance, the SGMT clade was clearly more dominant than the RO clade in impalas (Cersosimo et al., 2015), wallabies (May sample; Evans et al., 2009), in two separate studies involving Holstein dairy cows (Hook et al., 2009, 2011), in alpacas (St-Pierre and Wright, 2012), in water buffaloes (Franzolin et al., 2012), in sheep from Venezuela (Wright et al., 2008), in sheep from Scotland (Snelling et al., 2014), in New Zealand sheep fed two different diets (Seedorf et al., 2015), in Chinese roe deer (Li et al., 2014), and in reindeers (Norway and Svalbard; Sundset et al., 2009a,b). In contrast, the RO clade was distinctively more highly represented than the SGMT clade in the hoatzin (Wright et al., 2009), in an early analysis involving Holstein dairy cows (Whitford et al., 2001), in corn-fed beef cattle (Wright et al., 2007), in Jersey dairy cows (Skillman et al., 2006), and in horses fed a pasture or forage-grain diet (Fernandes et al., 2014). Notably, only a few studies have reported a balanced SGMT:RO, such as from potato-fed beef cattle (Wright et al., 2007).

**Table 1:** Representation of SGMT and R0 methanogens in different hosts and diets.

Host	SGMT^a^ (%)	RO^a^ (%)	Reference
Alpacas	70.0	17.6	St-Pierre and Wright (2012)
Bactrian camel (Potter sample)	30.2	66.0	Turnbull et al. (2011)
Bactrian camel (Southwick sample)	80.0	18.2	Turnbull et al. (2011)
Beef cattle (corn diet)	4.0	48.0	Wright et al. (2007)
Beef cattle (potato diet)	28.9	21.1	Wright et al. (2007)
Cattle (April 2010)^c^	38.0	49.0	Seedorf et al. (2015)
Cattle (September 2010)^d^	48.0	38.0	Seedorf et al. (2015)
Chinese roe deer (rumen)	77.0	1.0	Li et al. (2014)
Chinese roe deer (cecum)	68.0	1.0	Li et al. (2014)
Dairy cows (Holstein)	0.0	58.5	Whitford et al. (2001)
Dairy cows (Holstein)	65.7	32.5	Hook et al. (2009)
Dairy cows (Holstein)	93.4	5.9	Hook et al. (2011)
Dairy cows (Holstein)^b^	36.0	59.0	King et al. (2011)
Dairy cows (Jersey)	13.3	33.3	Skillman et al. (2006)
Dairy cows (Jersey)^b^	53.0	44.0	King et al. (2011)
Hoatzin	0.0	85.8	Wright et al. (2009)
Horses (pasture)^e^	0.0	66.3	Fernandes et al. (2014)
Horses (forage-grain)^e^	1.4	63.0	Fernandes et al. (2014)
Impalas	93.0	2.9	Cersosimo et al. (2015)
Sheep (lucerne)^f^	42.0	20.0	Seedorf et al. (2015)
Sheep (pasture)^g^	55.0	33.0	Seedorf et al. (2015)
Sheep (Scotland)	75.5–91.6	0.0–1.4	Snelling et al. (2014)
Sheep (Venezuela)	62.5	32.7	Wright et al. (2008)
Reindeer (Norway)	50.0	31.5	Sundset et al. (2009a)
Reindeer (Svalbard)	44.8	2.3	Sundset et al. (2009b)
Wallabies (May sample)	91.6	0.0	Evans et al. (2009)
Water buffaloes	62.5	28.1	Franzolin et al. (2012)

*^a^Representation presented as a percentage of the total methanogen population.*

*^b^Dairy cows of both breeds were maintained as a single herd under the same diet and environmental conditions.*

*^c^Values presented are the median of *n* = 15, as reported by Seedorf et al. (2015).*

*^d^Values presented are the median of *n* = 16, as reported by Seedorf et al. (2015).*

*^e^Values presented are the median of *n* = 6, as reported by Fernandes et al. (2014).*

*^f^Values presented are the median of *n* = 11, as reported by Seedorf et al. (2015).*

*^g^Values presented are the median of *n* = 8, as reported by Seedorf et al. (2015).*

In some reports, comparative studies have revealed opposite SGMT:RO population composition as a function of breeds or as a function of environmental factors within the same breed. This was observed in Holstein and Jersey dairy cows from the same herd maintained under common environmental conditions (King et al., 2011), as well as in cattle from New Zealand sampled at two different time points (Seedorf et al., 2015). In captive Bactrian camels sampled from zoological parks at two different locations in the USA, the SGMT:RO ratio for hindgut methanogens showed an opposite population structure pattern between the two sampled communities (Turnbull et al., 2011).

Dividing sequences between SGMT and RO clades can also help in uncovering differences in community structure between GIT samples that have a similar representation of *Methanobrevibacter*-related sequences. For instance, while they account for 93.0 and 85.8% of methanogens identified in sheep from Venezuela and in the hoatzin, *Methanobrevibacter*-related sequences have a completely opposite SGMT:RO distribution in these hosts. While additional studies are required to elucidate the respective contributions of host species genetics and environmental factors in the determination of whether the SGMT or the RO clade will be the most highly represented in a methanogen community, they may represent archaeal groups that thrive in different conditions. For instance, factors such as rumen or forestomach pH, tolerance to toxic compounds, and the rate of passage can act as selection agents, either individually or in combination, by promoting the growth of particular groups of methanogens, thereby affecting the population structure of the archaeal community (Janssen and Kirs, 2008). In this context, the natural division of *Methanobrevibacter*-like sequences into the SGMT and RO clades allows a higher level of specificity in developing population structure models for GIT methanogens that take into account phylogeny and representation, which can then be tested for methane production under controlled conditions *in vivo* or *in vitro*. This strategy could prove to be very valuable in the design of broad range mitigation strategies in the future.

### Other Methanogen Groups Commonly Identified in the GIT of Herbivores

In addition to *Methanobrevibacter*-related methanogens, other archaeal phylogenetic groups have also been frequently reported in herbivore GIT samples. Indeed, members of the order Methanomassiliicoccales (Iino et al., 2013), a group of methanogens also referred to as rice cluster III (Kemnitz et al., 2005), rumen cluster C (Janssen and Kirs, 2008) or Methanoplasmatales (Paul et al., 2012), are also a prominent group of GIT methanogens. Not only are they frequently found in GIT samples from livestock animals, they have also been found to be a highly prevalent type of archaea in the rumen environment. This has been the case in wallabies sampled in November (91.7%; Evans et al., 2009), sheep from Australia (80.8%; Wright et al., 2006), yak from China (79.4%; Huang et al., 2012), Svalbard reindeer (47.4%; Sundset et al., 2009b), and in beef cattle fed either a potato (50.0%) or corn (46.1%) diet (Wright et al., 2007). Rumen methanogens from this taxonomic group have been reported to use methylamines as substrates for methanogenesis (Poulsen et al., 2013). Since compounds such as betaine and choline have been shown to be metabolized by rumen bacteria to produce methylamines (Bradbeer, 1965; Neill et al., 1978; Mitchell et al., 1979; Moller et al., 1986; Eklund et al., 2005), their presence in certain feedstuffs such as molasses and wheat derived products, or their use as feed additives, may favor the prevalence of Methanomassiliicoccales methanogens in a rumen environment. Paul et al. (2012) also reported that uncultured Methanomassiliicoccales methanogens could be enriched from the gut of higher termites when methanol was used as a substrate for methanogenesis.

Since they have been found to be highly prevalent in host species that can also have a high representation of *Methanobrevibacter*-related methanogens, this information is necessary to generate more comprehensive models for methanogen populations in the GIT of herbivores, such as perhaps be incorporated with the SGMT-RO model. Interestingly, sequences from specific habitats tend to be associated with certain clades (Paul et al., 2012; Seedorf et al., 2014). However, the limited number of isolates or representative 16S rRNA gene sequences that are available may not currently allow the same level of resolution that can be obtained with *Methanobrevibacter*-related sequences (Seedorf et al., 2014).

While they are in general less abundant than *Methanobrevibacter*-related or Methanomassiliicoccales sequences, 16S rRNA gene sequences that are more closely related to other methanogen species, such as *Methanosphaera stadtmanae* and *Methanomicrobium mobile*, or genera, such as *Methanoculleus* and *Methanosarcina*, have also been identified in the GIT of herbivores. While they are usually detected at a low frequency, they have in some studies been shown to be the most prevalent methanogens under certain conditions. For instance, from studies conducted in India, 94.4% of 16S rRNA gene sequences identified in the rumen of Murrah buffaloes were closely related to *Methanomicrobium mobile* (Chaudhary and Sirohi, 2009), and abundances of 97.1 and 72.3% of the same methanogen group were reported in Surti buffaloes (Singh et al., 2011, 2013). Furthermore, archaea belonging to the order Methanomicrobiales were predominant in the GIT of Japanese local ponies and thoroughbred horses (Lwin and Matsui, 2014). It remains to be determined why these methanogens were so prevalent in these particular conditions while they are usually detected at a much lower frequency. *Methanosphaera stadtmanae* was found to be the most prevalent methanogen in the hindgut of captive orangutans (Facey et al., 2012). This methanogen species has a limited substrate range for methane synthesis, and is notably unable to use H_2_ and CO_2_ for this purpose. Digestion of fruit pectin in frugivores like the orangutan has been hypothesized to increase GIT concentrations of methanol and acetate, which would provide a favorable environment for *Methanosphaera stadtmanae* methanogens to thrive. Finally, *Methanocorpusculum labreanum* was found to be the most abundant (59.9%) in the hindgut of captive white rhinoceroses (Luo et al., 2013). The identification and predominance of this type of methanogen in a GIT environment is unusual compared to most other reported studies. Predominance of *Methanocorpusculum* has also been reported in the fecal microbiota of Irish Thoroughbred racehorses (O’Donnell et al., 2013), but, as pointed out by the authors of that study, the use of the 16S rRNA gene V4 region may have underestimated archaeal diversity. Another study on equine fecal microbiota found that *Methanocorpusculum*-related methanogens were co-abundant with *Methanobrevibacter*-related methanogens (Fernandes et al., 2014). *Methanocorpusculum* archaea were observed at a median of 17.7% in horses fed a forage-grain diet, and at a median of 31.9% in horses maintained on pasture. They were only found to be more abundant than *Methanobrevibacter*-related methanogens in samples collected 4 days after a transition from a forage-grain diet to pasture had occurred.

## Future Perspectives on GIT Methanogen Research in Herbivores

### Sequencing of GIT Methanogen Genomes

Progress in biological research is often the result of technological advancements that improve experimental approaches. Numerous investigations of GIT methanogen communities to date have been performed using denaturing gradient gel electrophoresis (DGGE) analyses or Sanger sequencing of clone libraries, which both have intrinsic limitations in scope and resolution. However, next-generation sequencing has greatly improved the scope of microbial ecology studies, providing more comprehensive sequence datasets as well as allowing analysis of more independent samples and replicates (Denman and McSweeney, 2015).

While great strides have been made in characterizing the taxonomic composition of rumen and GIT methanogen communities, there remains a critical need to further our understanding of their metabolism and cellular physiology, particularly for species or candidate species that tend to be the most abundant. This knowledge would greatly contribute to the development of practical mitigation strategies. By revealing the biochemical potential of an organisms through prediction of its proteome, genome sequencing represents an effective strategy to elucidate the physiology of poorly characterized organisms. In terms of methane mitigation, it could for instance allow the identification of enzymes whose activity may be targeted with chemical antagonists, or surface proteins that may be used as antigens for the production of antibodies. Whether the devised strategies directly target methanogenesis, aim at reducing growth rates of methanogens or antagonize interactions with other microorganisms, they each have the potential to reduce enteric methane production.

Representative genomes of methanogens that have been identified in the GIT of livestock are currently limited in number. For instance, *Methanobrevibacter ruminantium* (Leahy et al., 2010) and *Methanobrevibacter smithii* (Samuel et al., 2007) are the only GIT *Methanobrevibacter* for which genomic data and predicted proteomes have been described in peer-reviewed publications. Permanent drafts for *Methanobrevibacter boviskoreani* and *Methanobrevibacter wolinii* are available, while efforts to complete the genomes of *Methanobrevibacter millerae* and *Methanobrevibacter olleyae* are ongoing. Both *Methanobrevibacter gottschalkii* and *Methanobrevibacter thaurei* have been selected to have their genome sequenced [see the Joint Genome Institute (JGI), Genomes Online Database (GOD)^1^]. As discussed in previous sections, these methanogens together represent the most common or abundant GIT archaea in livestock animals.

For GIT intestinal methanogens belonging to the order Methanomassiliicoccales, three genomes have so far been reported, all from isolates cultured from human feces: *Methanomassiliicoccus luminyensis* (Dridi et al., 2012), *Methanomassiliicoccus alvus* (Borrel et al., 2012), and *Methanomassiliicoccus intestinalis* (Borrel et al., 2013). Once sequence information from Methanomassiliicoccales representatives isolated from livestock become available, it will be of great interest to compare their genome with the human isolates. Available genomes of methanogens that are generally less well represented in GIT environments include species from the genera *Methanosarcina* (Deppenmeier et al., 2002; Galagan et al., 2002; Maeder et al., 2006), *Methanosphaera* (Fricke et al., 2006), *Methanocorpusculum* (Anderson et al., 2009), and *Methanomicrobium mobile* (see JGI-GOD^1^).

Analysis of the *Methanobrevibacter ruminantium* genome is a good example of the information that can be obtained from predicting the proteome of a methanogen (Leahy et al., 2010). For instance, it revealed the ability to use formate in addition to H_2_ as a substrate for methanogenesis, showed that this organism is unable to synthesize coenzyme M, and provided a metabolic explanation for the requirement of acetate for growth. It also uncovered a large array of genes encoding putative adhesins, and identified loci related to phage genes. In addition, this genomic information can also be used as a reference for metagenomics and metatranscriptomics analyses in GIT environments.

While this technology is providing an unprecedented capacity for genome sequencing, as attested by the increasing number of published microbial genomes, the complete and accurate determination of a prokaryotic genome is not a trivial undertaking and requires research teams adept in technical and bioinformatic skills. In addition, an important limitation in this process is the isolation and cultivation of methanogens, which remain a challenge for many strains. Therefore, genomes to sequence should be strategically selected considering the wide diversity of methanogens that populate the GIT of herbivores. In this context, population structure studies such as summarized in this review that are based on representation and phylogeny provide a critical basis in the selection of methanogens of interest.

In the long term, providing an increased number of available GIT methanogens genomes is essential for the development of effective and comprehensive mitigation strategies. Since the use of entire genome sequences dramatically improves phylogenetic analysis of archaea compared to only using 16S rRNA gene sequences (Brochier-Armanet et al., 2011), this will allow the accurate identification of phylogenetic nodes that are shared by clusters of GIT methanogens, which can be targeted for mitigation. In addition, comparative genome analyses will reveal conserved proteins within phylogenic clusters of methanogens, such as surface molecules that can be targeted by vaccination or intracellular factors that can be targeted for chemical inhibition. Alternatively, metatranscriptomics can also be used to identify mitigation targets. For instance, Shi et al. (2014) recently reported that transcription of methanogenesis pathway genes was elevated in sheep with high methane emissions.

### Culture-Based Investigations of GIT Methanogen Microbiology

As highlighted in the previous section, the available community compositions from gut methanogens in herbivores has revealed that, while there can be some overlap between samples, each so far appears to be unique. By mechanisms that are currently unknown, certain methanogens can be prevalent under particular conditions (e.g., host breed, species, diet, or geographical location), while they are detected at a lower frequency in other cases. In order to gain further insight, there needs to be an increase in culture-based microbiological studies of GIT methanogens, which are better suited for mechanistic studies that require a controlled environment. Due to the limited number of GIT methanogen species that have successfully been isolated and grown *in vitro* (Creevey et al., 2014), direct culturing of GIT samples represents an attractive alternative which would yield valuable insights not only about methanogens, but also of their interactions with other members of the community. The importance of such investigations can be emphasized by reports such as by Popova et al. (2013), where differences in methane production capacity were found between rumen and cecal contents from lambs fed high grain content diets, despite *Methanobrevibacter*-related methanogens being the most abundant archaea in both environments.

### Investigation of Intra-Community Interactions Involving Methanogens

The complexity of GIT microbial communities in herbivores is not simply due to their high cellular density and diversity, but is also a result of intricate networks of inter-species trophic relationships. Methanogens depend on other microorganisms for substrates such as H_2_ and CO_2_ to sustain their energy needs through anaerobic respiration and methane synthesis. While methanogens can acquire substrates from their surrounding environment, some can associate intimately with protozoa or fungi. For instance, it was reported that the free-living (FL) and protozoa-associated methanogen (PAM) populations were composed of the same major groups (*Methanobrevibacter* and *Methanomicrobium*), but that their composition differed between FL and PAM (Tymensen et al., 2012). In addition, the distribution of species-level OTUs within the same subgroups was found to differ as well. A study by Belanche et al. (2014) also reported that PAMs represented a more variable population than FL methanogens. If such interactions contribute to greater methane production, then their disruption could potentially be used as a mitigation strategy. It will also be of interest to investigate the degree of specificity between partner species that is required for these cell–cell interactions to occur.

The potential of specific trophic relationships between methanogens and bacteria should also be further explored. In studies conducted in sheep as a model ruminant (Morgavi et al., 2012), it was reported that the liquid-associated bacterial and methanogens fraction of animals kept without protozoa for more than 2 years produced more methane than the corresponding rumen fractions from faunated animals or animals defaunated for only a few months. Accordingly, the same study found that animals maintained without protozoa for more than 2 years were higher methane emitters than animals that had been defaunated for a few months.

## Concluding Remarks

While great strides have been made in the study of rumen methanogen populations in a variety of hosts and environmental conditions, further investigations still are required in order to gain sufficient insight to develop comprehensive methane mitigation strategies targeting methanogens. It will only be through a sustained effort in combining genomics and cellular analyses that this goal may be reached in the near future.

## Conflict of Interest Statement

The authors declare that the research was conducted in the absence of any commercial or financial relationships that could be construed as a potential conflict of interest.

## Footnotes

1 https://gold.jgi-psf.org/
